# Retraction Note: Inhibition of autophagic flux differently modulates cannabidiol-induced death in 2D and 3D glioblastoma cell cultures

**DOI:** 10.1038/s41598-024-51877-z

**Published:** 2024-01-16

**Authors:** Vladimir N. Ivanov, Peter W. Grabham, Cheng-Chia Wu, Tom K. Hei

**Affiliations:** Center for Radiological Research, Department of Radiation Oncology, Vagelos College of Physicians and Surgeons, Columbia University, New York, NY 10032 USA

Retraction of: *Scientific Reports* 10.1038/s41598-020-59468-4, published online 14 February 2020

The Editors have retracted this Article.

This follows an investigation by Columbia University into the research practices of members of this authorship group. Amongst its findings, this investigation concluded that the content of images in 4f and 4g is inconsistent with the raw data. Specifically:

• In the published Figure 4f, lanes 2, 3 and 4 are described as having CBD at three concentrations (10, 20 and 20 μM). However, in the raw data, the annotation shows that only lanes 3 and 4 contained CBD.

• In the published Figure 4g, none of the experimental conditions include Gy (“Gray,” a unit of radiation), and there is no mention in the manuscript that these cells were irradiated. However, in the raw data the annotations show that all four lanes were irradiated (noted with “Gy”). Additionally, the published figure indicates that the cells were treated with a STAT3 inhibitor-6 (STAT3_i6), which is inconsistent with the annotations in the raw data.

• Lane 2 in the published image for 4g contains CBD, whereas lane 2 in the full blot viewed in the raw data does not.

The Editors therefore no longer have confidence in the results and conclusions presented.

Peter W. Grabham, Cheng-Chia Wu and Tom K. Hei agree with the retraction and its wording. Vladimir N. Ivanov disagrees with the retraction.

